# Loss of maternal calcitriol reversibly alters early offspring growth and skeletal development in mice

**DOI:** 10.1093/jbmr/zjae035

**Published:** 2024-03-04

**Authors:** Sarah A Hartery, Beth J Kirby, Emma C Walker, Martin Kaufmann, Glenville Jones, René St-Arnaud, Natalie A Sims, Christopher S Kovacs

**Affiliations:** Faculty of Medicine – Endocrinology, Memorial University of Newfoundland, St. John’s, Newfoundland, A1B 3V6, Canada; Faculty of Medicine – Endocrinology, Memorial University of Newfoundland, St. John’s, Newfoundland, A1B 3V6, Canada; St. Vincent’s Institute of Medical Research, the University of Melbourne, Melbourne, 3065, Australia; Department of Biomedical and Molecular Sciences, Queen’s University, Kingston, Ontario, K7L 3N6, Canada; Department of Biomedical and Molecular Sciences, Queen’s University, Kingston, Ontario, K7L 3N6, Canada; Shriners Hospitals for Children – Canada and McGill University, Montréal, Quebec, H4A 0A9, Canada; St. Vincent’s Institute of Medical Research, the University of Melbourne, Melbourne, 3065, Australia; Faculty of Medicine – Endocrinology, Memorial University of Newfoundland, St. John’s, Newfoundland, A1B 3V6, Canada

**Keywords:** calcitriol, Cyp27b1, 1α-hydroxylase, neonate, phosphorus, calcium, parathyroid hormone, calcitriol, skeleton, mineralization

## Abstract

Ablation of *Cyp27b1* eliminates calcitriol but does not disturb fetal mineral homeostasis or skeletal development. However, independent of fetal genotypes, maternal loss of *Cyp27b1* altered fetal mineral and hormonal levels compared to offspring of WT dams. We hypothesized that these maternal influences would alter postnatal skeletal development. *Cyp27b1* null and WT females were mated to bear only *Cyp27b1*^+/−^ offspring. Forty-eight hours after birth, pups were cross-fostered to dams of the same or opposite genotype that bore them. Maternal and offspring samples were collected on days 21 (weaning) and 42. Offspring measurements included minerals and hormones, BMC by DXA, ash weight and mineral content, gene expression, 3-point bending tests, and microCT. Maternal lactational behavior was evaluated. Milk was analyzed for nutritional content. At day 21, offspring fostered by nulls, independent of birth dam, had ~20% lower weight, BMC, ash weight, and ash calcium than pups fostered by WT dams. Adjustment for body weight accounted for the lower BMC but not the lower ash weight and ash calcium. Hormones and serum/urine minerals did not differ across offspring groups. Offspring fostered by nulls had shorter femurs and lower cortical thickness, mean polar moment of inertia, cortical area, trabecular bone volume, and trabecular number. Dam lactational behaviors and milk nutritional content did not differ between groups. At day 42, body weight, ash weight, lengths, BMC, and tibial bone strength were no longer different between pups fostered by null vs WT dams. In summary, pups fostered by *Cyp27b1* nulls, regardless of birth dam, have proportionately smaller skeletons at 21 d, impaired microstructure, but normal mineral homeostasis. The skeletal effects are largely recovered by day 42 (3 wk after weaning). In conclusion, maternal loss of calcitriol impairs early postnatal cortical bone growth and trabecular bone mass, but affected offspring catch up after weaning.

## Introduction

Calcitriol is critical for normal postnatal mineral and skeletal homeostasis. Human and animal studies have shown that severe deficiency of vitamin D, as well as inactivating mutations of the genes encoding 1ɑ-hydroxylase (*CYP27B1)* or the vitamin D receptor (*VDR*), cause reduced intestinal mineral absorption, hypocalcemia, hypophosphatemia, secondary hyperparathyroidism, and under-mineralized skeletons (rickets or osteomalacia, depending upon age of onset).^1^ Calcitriol’s primary action is to stimulate intestinal mineral delivery, through which calcitriol indirectly promotes bone formation and mineralization. Animal models have confirmed that selective ablation of *Vdr* from intestinal cells, or dietary calcium deficiency, produces the same phenotype as severe vitamin D deficiency or global absence of *Vdr.*^2^ Conversely, the rachitic phenotype from global loss of *Vdr* can be rescued by restoring *Vdr* expression to intestinal cells.^2^^,^^3^

During fetal development, normal human cord and murine fetal blood typically show low calcitriol, 25OHD concentrations of about 75%–100% of simultaneous maternal values, proportionately higher concentrations of 24-hydroxylated and C-3-epimerized vitamin D metabolites, increased serum calcium and phosphorus, low PTH, and low to normal intact FGF23.^4–6^ The bulk of evidence from human and animal studies indicates that calcitriol is not required for fetal mineral homeostasis, skeletal development, or skeletal mineralization.^4^ At birth, severely vitamin D-deficient human babies have normal cord blood calcium, phosphorus, PTH, skeletal morphology, and skeletal mineral content.^4^ Human babies bearing homozygous or compound heterozygous inactivating mutations of *CYP27B1* or *VDR* are also normal at birth.^4^^,^^7–18^ Serum calcium, phosphorus, PTH, and the endochondral skeleton are also normal in severely vitamin D-deficient fetal rats,^19–21^ *Vdr* null fetal mice,^22^^,^^23^ and *Cyp27b1* null fetal mice.^24^

It is after birth, when intestinal calcium and phosphate absorption become dependent on calcitriol, that severe vitamin D deficiency or loss of *CYP27B1* or *VDR* in humans may cause hypocalcemia, followed by the development of skeletal rickets over succeeding months, with a peak incidence in the second year.^4^^,^^7–18^^,^^25–27^ Similarly, in animal models, it is after weaning that hypocalcemia, hypophosphatemia, secondary hyperparathyroidism, and rickets develop.^19^^,^^20^^,^^28–31^ In humans with inactive VDRs, and in *Vdr* null and *Cyp27b1* null neonatal mice, a diet enriched in calcium, or parenteral use of calcium, maintains normal mineral metabolism and prevents rickets.^4^

In the earlier human and animal studies, the effects of disorders intrinsic to the fetus have been examined, whereas the effects of disrupted maternal vitamin D physiology on the fetus have been less clearly discriminated. We recently reported that *Cyp27b1* null fetal mice, which do not make calcitriol, had normal serum calcium and phosphate, PTH, FGF23, skeletal mineral content, tibial lengths and morphology, and placental calcium transport.^24^ When we directly compared fetal littermates of *Cyp27b1* null dams, they were biochemically and phenotypically indistinguishable from each other, despite their differing genotypes. This was also true for fetal littermates of related WT dams. However, when comparing fetuses of *Cyp27b1* null and WT sisters, fetuses from *Cyp27b1* null dams had higher serum and amniotic fluid calcium, lower amniotic fluid phosphorus, lower FGF23, and higher 25OHD and 24,25-dihydroxyvitamin D (24,25(OH)_2_D). These findings revealed that although loss of fetal calcitriol did not alter mineral or bone homeostasis, loss of maternal calcitriol did alter fetal mineral homeostasis independently of fetal genotype.

The current studies were undertaken to determine if maternal genotype influenced mineral homeostasis and skeletal development in the offspring after birth. To do this, we cross-fostered pups from *Cyp27b1* null and related WT dams shortly after birth and examined them at 3 and 6 wk of age. This enabled us to analyze for effects of maternal calcitriol sufficiency vs deficiency on the resulting offspring phenotype.

## Materials and methods

### Animal husbandry

The engineering of the *Cyp27b1* deletion model has been previously described.^29^ *Cyp27b1* mice have been maintained in a C57BL/6 background by breeding heterozygous-deleted mice together, and periodically back-crossing into the parent strain. Genotyping was done by PCR on DNA using previously described primer sequences.^29^

WT and *Cyp27b1* null mice, first-degree relatives of each other, were maintained on a “rescue diet” containing 2% calcium, 1.25% phosphorus, and 20% lactose (Envigo-TekLad TD94112, Envigo RMS). The diet’s high lactose content increases paracellular calcium absorption, and is a standard in prior studies of *Cyp27b1* null and *Vdr* null mice to normalize fertility.^32–34^ Pups born of these experimental mice were switched to regular 1% calcium chow at the time of weaning (Envigo-Teklad TD2018).

**Figure 1:**
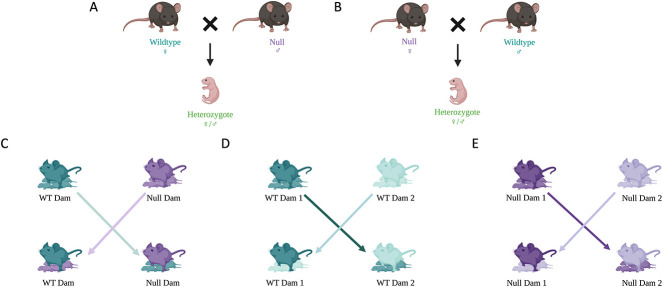
Mating and cross-fostering schemes. (A) WT dams were mated to *Cyp27b1* null males, while in B, *Cyp27b1* null dams were mated to WT males. All dams bore only *Cyp27b1^+/−^* pups. (C) On day 2 after birth, pups born of WT dams were cross-fostered to *Cyp27b1* null dams and vice versa. The differing colors of dams and pups reflect that pups have been cross-fostered to dams of the opposite genotype from the birth dams. (D and E) Controls included cross-fostering pups of WT dams to WT dams, and pups of *Cyp27b1* null dams to *Cyp27b1* null dams. The similar colors of dams and pups reflect that the pups have been cross-fostered to dams of the same genotype as the birth dams. Images created with BioRender.com.

WT females were mated to *Cyp27b1* null males, while *Cyp27b1* null females were mated to WT males (all mice from the same colony) such that all dams bore only *Cyp27b1*^+/−^ offspring (Figure 1A and B). This was done so that each dam would experience uniform demands for mineral delivery from the same numbers of offspring of a single genotype, thereby eliminating variability from dam to dam that would be caused by different fetal and neonatal genotypes. Pairs of mice were mated on the same nights, and the presence of a vaginal mucus plug the next morning marked embryonic day 0.5. Normal gestation is 19 d.

The Institutional Animal Care Committee of Memorial University of Newfoundland approved all procedures involving live animals.

### Cross-fostering and sample collection

Forty-eight hours after birth, all pups were cross-fostered to dams that delivered concurrently (Figure 1C–E). To prevent rejection of pups, they were placed into the cage of the foster dam after she was temporarily removed. Bedding and urine from the foster dam were rubbed over the pups before introducing her to the cross-fostered pups. Litter sizes were adjusted if needed to maintain a mean of 7 pups per dam; no pups were lost from maternal rejection of the cross-fostered litters. The 4 groups included pups of WT dams fostered by *Cyp27b1* nulls (WT → null), pups of *Cyp27b1* null dams fostered by WT (null→WT), and 2 controls (WT → WT and null→null). As noted above, all offspring were *Cyp27b1*^+/−^.

Maternal and offspring samples were collected on day 21 (normal weaning) and 42 (3 wk post-weaning). Offspring urine was collected in a clean dish before blood was collected by heart puncture. The pups were then euthanized by cervical dislocation. Kidneys and duodena were harvested, snap-frozen in liquid nitrogen, and stored at −80°C. Pup bodies were sexed by genital exam, weighed, measured, scanned for bone mineral content, and stored at −20°C. Adult dams promptly voided in a clean cage. Blood was then obtained by cardiac puncture, after which the mouse was euthanized by rapid cervical dislocation.

### Anthropometry

Pups were weighed and measured from nose to base of tail.

### Milk collection

Milk was collected on day 10. Dams were separated from their pups for 2 h and then given 1 IU of oxytocin intraperitoneally. Pups were kept warm in their cage in the meantime. Twenty minute after injection of oxytocin, with the dam supine and anesthetized, all 10 mammary glands were gently massaged from base to tip to express milk, which was collected using a pipette. Milk was stored at −20°C until analyzed.

### Chemical and hormone assays

Serum or plasma samples were separated as previously described.^35^^,^^36^ Individual samples were pooled if the sample size requirements of an assay exceeded the recoverable blood volume. Calcium, phosphorus, and magnesium were analyzed using colorimetric assays (Sekisui Diagnostics PEI Inc.). Enzyme-linked Immunosorbent Assays (EIAs) measured intact PTH (Immutopics), calcitriol (Immunodiagnostic Systems Ltd), intact FGF23 (Kainos), and insulin growth factor type I (IGF-I; R&D Systems). Any values below the assay sensitivity were reset to values equal to the detection limit. For the calcitriol assay, this is 6 pmol/L in human sera but likely 20 pmol/L based on our prior work in mouse sera.^37^

25OHD_3_, 24,25(OH)_2_D_3,_ 25-OH-D_3_-26,23-lactone, and 3-epi-25-OH-D_3_ were assayed by liquid chromatography–tandem mass spectrometry (LC–MS/MS) as previously detailed.^38^

Milk component measurements included triglycerides by colorimetric assay (Sekisui Diagnostics), protein using the Pierce Protein Assay Kit 23 227 (ThermoFisher Scientific), and calcium by atomic absorption spectroscopy as described previously.^39^ Samples of 20 μL of milk were drawn into capillary tubes and centrifuged for 20 min to determine the creamatocrit (proportion of cream to total volume), fat concentration, and energy value.^40^ Moisture content was assessed by weighing 20 μL of liquid milk, drying the samples at 60°C for 24 h, and then measuring the dry weights.^41^

### Offspring ash and skeletal mineral assay

As previously described,^42^ intact pups at day 21 and 42 were reduced to ash in a furnace (500°C × 24 h). Each day 21 ash sample was dissolved in 1.5 mL of nitric acid and 57.7 mL of water. A Perkin Elmer 2380 Atomic Absorption Flame Spectrophotometer assayed the calcium and magnesium content of the ash, while the phosphorus content was determined using the colorimetric assay (Sekisui Diagnostics).

### Bone mineral content

A PIXImus 2 Bone Densitometer (GE Lunar), calibrated daily to a standard phantom, was used to obtain whole body DXA measurements of the offspring at days 21 and 42.

### Bone harvest, biomechanical testing, and MicroCT

Tibias and femora were harvested at days 21 and 42 and stripped of soft tissues.

One tibia from each mouse was stored at −20°C and later rehydrated in phosphate-buffered saline at room temperature for at least 2 h. Cortical bone strength was assayed using a single column Instron Series 3340 electromechanical test instrument (Instron), as previously described.^43^

One femur from day 21 was fixed in 10% buffered formalin and analyzed using a high resolution microCT scanner (Skyscan 1276), using previously described methods,^43^ with the following modifications: software versions used were NRecon (version 1.7.4.2), DataViewer (version 1.5.4), and CT Analyzer (version 1.17.7.2). Trabecular bone was measured in a region commencing at 6% of each femur’s length from the growth plate, with a region of interest of 6% femur length extending toward the femoral mid-shaft, with a lower threshold equivalent to 0.37198 g/cm^3^ calcium hydroxyapatite (CaHA). The lower threshold for detecting cortical bone was equivalent to 0.51362 g/cm^3^ CaHA.

### Surveillance of maternal behavior

Dams and their litters were recorded at time of cross-fostering (48 h after birth) and on day 10 (following milk collection). Surveillance extended over 3 d from a Friday to a Monday for a total of 6 d. The facility is on a controlled 12-h light/12-dark h cycle, which facilitated recording of each dam’s daylight and nocturnal behavior.

Cameras from the Blink home security camera system (Amazon) were positioned to capture activity in the nest and the rest of the cage. The cameras were remotely controlled; red status lights were disabled so as not to disturb the dams. Cameras were set to motion detection mode and recorded 30-s video clips when activated. Recordings were downloaded for later analysis.

The following phases classified each dam’s behavior: active but off the litter (not nursing); active on the litter (nursing but pups might not be able to latch easily); non-active on the litter (resting on the litter making it easiest for pups to latch). Proportion of time spent in the 3 different phases was averaged for each dam from the 2 different assessment intervals.

### RNA extraction and real-time quantitative RT-PCR

Offspring kidneys and duodena were snap-frozen in liquid nitrogen. Total RNA was purified using the RNeasy Midi Lipid kit (Qiagen). RNA quantity and quality were confirmed with the Agilent 2100 BioAnalyzer (Agilent Technologies). We used TaqMan® Gene Expression Assays (with the manufacturer’s pre-designed primers and probes for optimal amplification) and Fast Advanced Master Mix from Applied BioSystems (ABI)/Life Technologies to determine the expression of *Ca^2+^-ATPase* (*Pmca1*), *Calbindin-D9k* (*S100g*), *Trpv6*, *Slc8a1*, *and Kcnma1*. The reference gene used for these studies was *Gapdh*. Details of conditions and cycle times have been previously reported.^39^^,^^44^^,^^45^ Briefly, cDNA was synthesized using the High Capacity cDNA Reverse Transcription Kit (ABI), and multiplex quantitative PCR (qPCR) reactions were run in triplicate on the ViiA 7 Real-Time PCR System (ABI).^22^^,^^44^ The minimum sample size was 5 for each genotype. Relative expression was determined from the threshold cycle (C_T_) normalized to the reference gene.

### Statistical analysis

Data were analyzed using GraphPad Prism 10 for MacOS, Version 10.0.3 (217) (GraphPad Software). Two-way ANOVA followed by Šidák multiple comparisons test was used to analyze the 4-group offspring data and reveal differences attributable to foster dam versus birth dam. Maternal data were analyzed in 2 groups (*Cyp27b1* null vs WT) by *t*-test. qPCR data were analyzed by the Comparative C_T_ Method (2^ΔCT^).^46^ All data are expressed as mean ± SD. Although not part of the initial study design, a post hoc analysis segregated by sex was conducted; these data are shown in the Supplementary Figures.

## Results

### Altered skeletal phenotype of pups fostered by Cyp27b1 nulls

**Figure 2:**
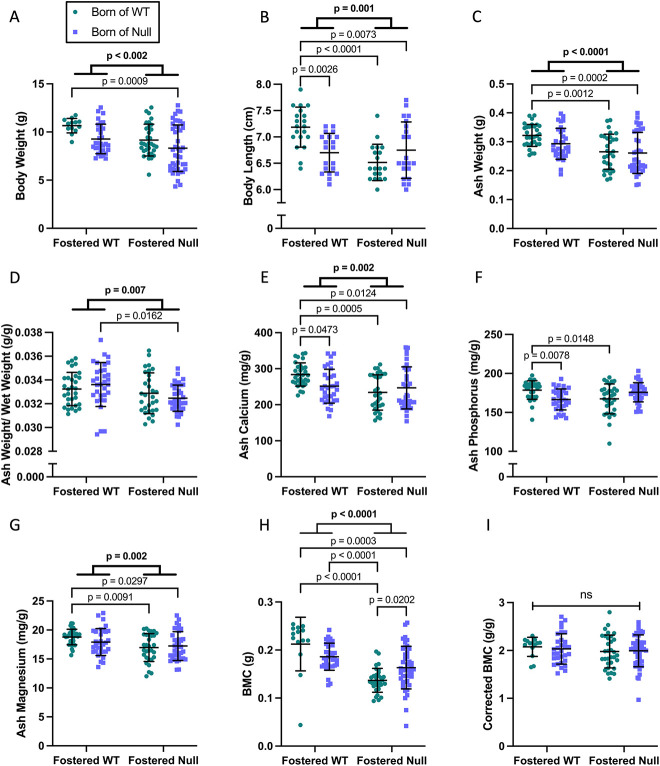
Skeletal parameters of offspring at day 21. Pups fostered by *Cyp27b1* null dams had lower body weight (A), length (B), and ash weight (C); the difference in ash weight was attenuated but remained significant when corrected for body weight (D). Ash calcium and magnesium were reduced in pups fostered by *Cyp27b1* nulls, while ash phosphorus content was not (EFG); however, ash calcium and phosphorus content were both lower in null→WT as compared to WT → WT pups. Whole body BMC was reduced in pups fostered by *Cyp27b1* null dams (H), but the difference was eliminated after adjustment for body weight (I). Significant subgroup differences included that null→null pups had significantly lower body weight, body length, ash weight, ash calcium, ash magnesium, and BMC, than WT → WT pups. Data are shown as individual values, with mean and standard deviation. *P*-values are from 2-way ANOVA (bold) with Šidák post hoc tests.

At the normal time of weaning (day 21), pups fostered by null dams showed growth restriction with significantly lower body weights (Figure 2A), shorter body lengths (Figure 2B), and lower ash weights as compared to pups fostered by WT dams (Figure 2C). Adjustment for body weight attenuated but did not eliminate the difference in ash weight (Figure 2D). Ash calcium and magnesium content per gram of ash were reduced in pups fostered by nulls, while phosphorus content was no different between groups (Figure 2E–G). Whole body BMC (by DXA) was reduced in pups fostered by null dams (Figure 2H), but the difference was eliminated after adjustment for pup weight (Figure 2I). Overall, the findings indicate growth restriction in pups fostered by null dams, regardless of their birth dam, with a proportionate reduction in skeletal size, and disproportionate under-mineralization of bone. A sex-specific analysis showed the same lower body weight, length, and BMC in male and female pups fostered by null dams, with no differences in BMC after correction for body weight (Supplementary Figure 1).

**Figure 3:**
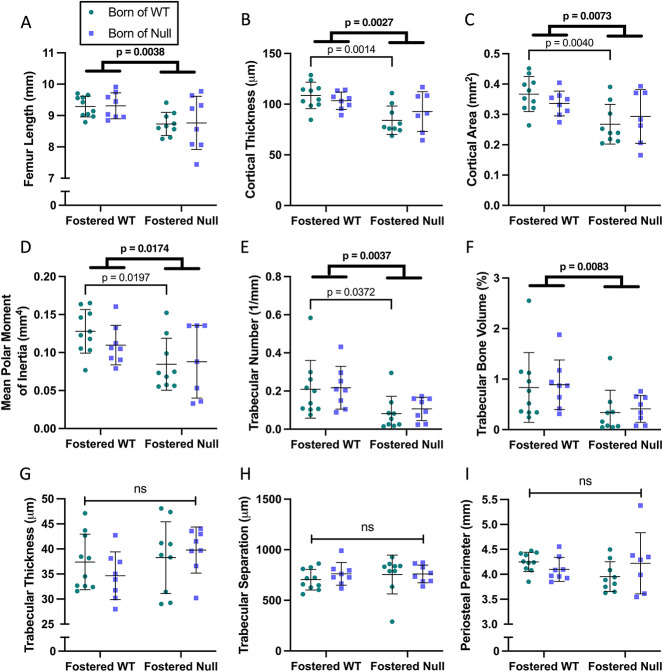
MicroCT analysis of offspring femora at day 21. Femora of pups fostered by *Cyp27b1* null dams had lower femur lengths (A), cortical thickness (B), cortical area (C), and mean polar moment of inertia (D). Trabecular number (E) and trabecular bone volume (F) were reduced, while there were no significant differences in trabecular thickness (G), separation (H), or periosteal perimeter (I). Data are shown as individual values, with mean and standard deviation. *P*-values are from 2-way ANOVA (bold) with Šidák post hoc tests.

MicroCT of femora revealed that pups fostered by null dams had shorter femurs (Figure 3A), accompanied by lower cortical thickness, cortical area, and mean polar moment of inertia (Figure 3B–D). Trabecular number and bone volume were lower (Figure 3E and F), while there were no significant differences in trabecular thickness, trabecular separation, or periosteal perimeter (Figure 3G–I). The difference in trabecular number without any change in trabecular separation likely relates to the very low trabecular bone volume and small tibial size at this age which imposes an artificial upper limit on this parameter, thereby blunting any increase that might have occurred. The observed effects were attributable to the foster dam and not the birth dam. A sex-specific analysis showed preservation of the relative changes seen in Figure 3, but the results were statistically significant only in female mice (Supplementary Figure 2).

**Figure 4:**
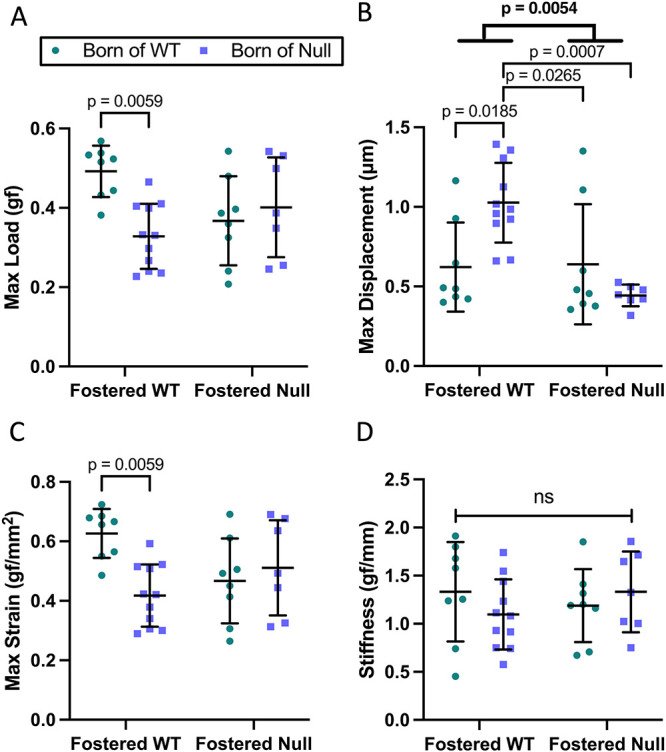
Biomechanical testing of offspring tibias at day 21. Three weeks after birth, a 3-point bend test indicated a higher ultimate load in WT→WT tibias (A), higher displacement in null→WT tibias and in tibias of pups fostered by WT vs fostered by *Cyp27b1* null dams (B), increased maximum strain in WT → WT tibias (C), and no difference in stiffness across groups (D). The tibias broke easily and many bent rather than breaking, as described in the Results section. Data are shown as individual values, with mean and standard deviation. *P*-values are from 2-way ANOVA (bold) with Šidák post hoc tests.

Biomechanical testing was problematic on day 21 tibias. A similar number from each group broke (1 WT → WT; 2 null→WT; 1 WT → null; 3 null→null) during handling, while multiple other bones bent rather than breaking during the test (6 null→WT; 1 WT → null). The device recorded the bones as having broken at the point they simply bent. Using data from tibias that broke or bent revealed reduced bone strength (maximum load and strain) in all tibias compared to those from WT → WT. Increased displacement in the null→WT tibias reflected that most bent rather than breaking, and no difference in stiffness (Figure 4A–D). Overall, at day 21, the tibias were insufficiently mineralized for this test to be reliably carried out, but an effect of both foster dam and birth dam is suggested by the data. A sex-specific analysis preserved the relative changes seen in Figure 4, with between-dam statistical significance retained only for reduced displacement in male pups fostered by null dams (Supplementary Figure 3).

### Biochemical and hormonal indices of pups

**Figure 5:**
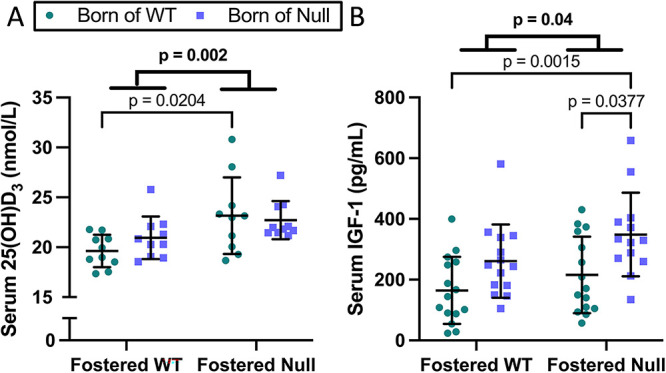
Serum calcifediol and IGF-I in pups at day 21. Serum 25(OH)D_3_ was modestly but significantly higher in pups fostered by *Cyp27b1* null dams as compared to pups fostered by WT dams (A). Serum IGF-I was higher in pups fostered by null dams vs fostered by WT dams; there was also a birth dam effect with lower IGF-I in WT→WT pups as compared to null→null pups (B). Data are shown as individual values, with mean and standard deviation. *P*-values are from 2-way ANOVA (bold) with Šidák post hoc tests.

There were no significant differences in serum or urine minerals (Table 1). Among hormonal indices, there were no significant differences in PTH, FGF23, calcitriol, or vitamin D metabolites (24,25(OH)_2_D_3,_ 25-OH-D_3_-26,23-lactone, 3-epi-25-OH-D_3_, 25(OH)D_3_:24,25(OH)_2_D_3_ ratio, and 25(OH)D_3_:lactone ratio) (Table 1). Serum 25(OH)D_3_ was modestly but significantly higher in pups fostered by null dams as compared to pups fostered by WT dams (Figure 5A). Serum IGF-I showed higher levels in pups fostered by null dams; IGF-I was also significantly lower in WT → WT pups as compared to null→null pups, consistent with an effect of the birth dam (Figure 5B).

**Table 1:** Serum and urine minerals and serum hormones in pups at day 21.

	**WT→WT**	**NULL→WT**	**WT→NULL**	**NULL→NULL**	***P*-values**
**SERUM**					
Calcium (mmol/L)	2.11 ± 0.04 (15)	1.96 ± 0.06 (15)	1.93 ± 0.04^a^ (15)	2.08 ± 0.06 (15)	NS
Phosphorus (mmol/L)	3.87 ± 0.11 (15)	4.01 ± 0.14 (15)	4.06 ± 0.20 (15)	3.89 ± 0.15 (15)	NS
PTH (pg/mL)	42.98 ± 5.03 (15)	64.64 ± 12.54 (15)	47.67 ± 5.30 (15)	54.32 ± 9.12 (15)	NS
FGF-23 (pg/mL)	111.57 ± 12.40 (15)	122.40 ± 13.97 (15)	99.16 ± 15.96 (15)	146.11 ± 16.58 (15)	NS
Calcitriol (pmol/L)	240.06 ± 64.16 (14)	193.35 ± 51.68 (14)	276.87 ± 74.00 (14)	283.87 ± 75.87 (14)	NS
24,25(OH)_2_D_3_ (ng/mL)	2.40 ± 0.54 (10)	2.59 ± 0.89 (10)	2.15 ± 0.64 (10)	2.80 ± 1.13 (10)	NS
25-OH-D_3_-26,23-lactone (ng/mL)	1.60 ± 0.31 (10)	1.52 ± 0.39 (10)	1.54 ± 0.38 (10)	1.72 ± 0.44 (10)	NS
3-epi-25-OH-D_3_	2.23 ± 0.40 (10)	2.58 ± 0.62 (10)	2.98 ± 1.76 (10)	2.71 ± 0.56 (10)	NS
25(OH)D_3_:24,25(OH)_2_D_3_ ratio	3.39 ± 0.56 (10)	3.67 ± 1.35 (10)	4.53 ± 0.80 (10)	3.73 ± 1.33 (10)	NS
25(OH)D_3_:lactone ratio	5.11 ± 1.17 (10)	5.98 ± 2.27 (10)	6.25 ± 1.23 (10)	5.57 ± 1.29 (10)	NS
**URINE**					
Calcium/Creatinine (mmol/mmol)	6.85 ± 1.00 (14)	6.13 ± 0.88 (15)	6.53 ± 1.81 (20)	4.27 ± 0.87 (19)	NS
Phosphorus/Creatinine (mmol/mmol)	30.70 ± 7.12 (12)	20.12 ± 3.53 (13)	27.48 ± 3.86 (16)	36.66 ± 5.03 (17)	NS
Magnesium/Creatinine (mmol/mmol)	14.81 ± 1.37 (15)	11.23 ± 1.26 (16)	10.13 ± 1.63 (20)	9.99 ± 1.71 (19)	NS

All values are mean ± SD; the numbers of samples are indicated in parentheses. ^a^*P* < .08 vs. WT → WT. All other *P*-values are *P* > .1 and therefore not significant (NS).

### Duodenal and renal gene expression

**Figure 6:**
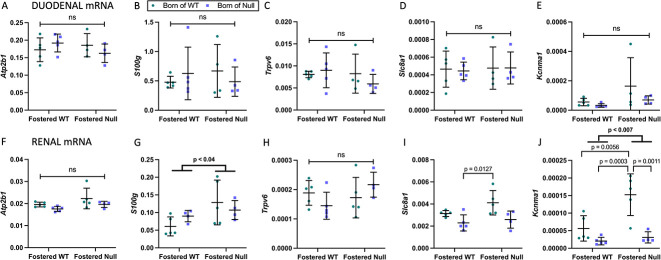
Gene expression in pup duodena and kidneys at day 21. Offspring duodena showed no significant changes in the mRNA levels of *Atpb2* (A), *S100g* (B), *Trpv6* (C), *Slc8a1* (D), and *Kcnma1* (E). Offspring kidneys similarly showed no significant changes in the mRNA levels of *Atpb2* (F), *S100g* (G), *Trpv6* (H), whereas small but statistically significant changes in *Slc8a1* (I) and *Kcnma1* (J) mRNA levels were likely not physiologically important given their very low levels of expression. Data are shown as individual fold values relative to the housekeeping gene, with mean fold and standard deviation. *P*-values are from 2-way ANOVA (bold) with Šidák post hoc tests.

Duodena and kidneys of the offspring were assessed for changes in calciotropic gene expression. There were no significant differences in duodenal mRNA levels of *Atpb2*, *S100g*, *Trpv6*, *Slc8a1*, and *Kcnma1* (Figure 6A–E). In the kidneys, there were no significant differences in *Atpb2*, *S100g*, and *Trpv6* (Figure 6F–H). Small, statistically significant changes in *Slc8a1* and *Kcnma1* mRNA levels were observed, but given their very low levels of expression, these may not be physiologically important (Figure 6I and J).

### Maternal parameters

**Figure 7:**
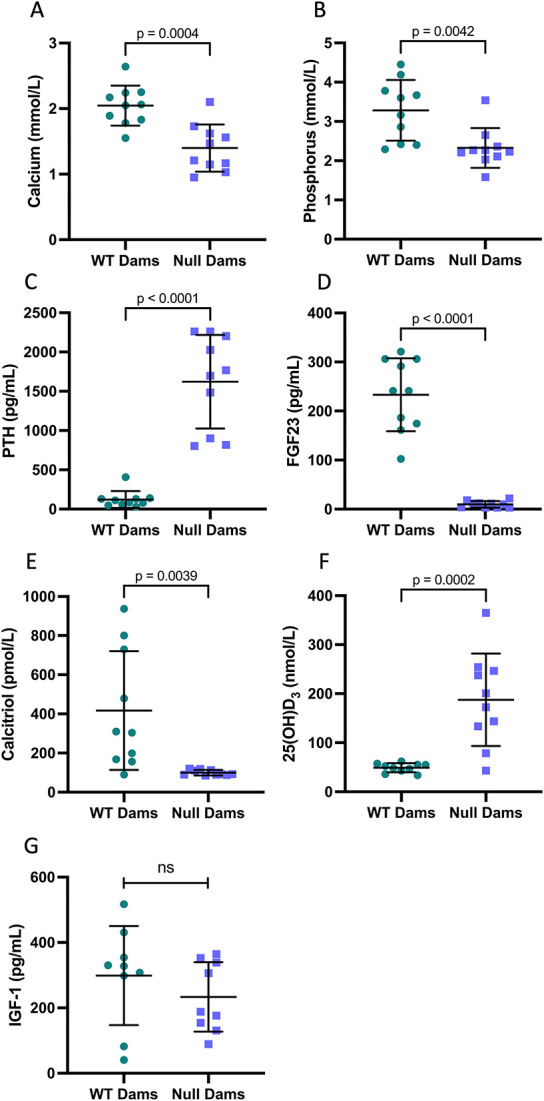
Maternal parameters at day 21. Consistent with our early study, *Cyp27b1* null females were hypocalcemic (A) with low serum phosphorus (B), increased PTH (C), lower FGF23 (D), low calcitriol (E), and higher 25(OH)D_3_ (F). IGF-I was variable with no significant differences among the groups (G). Data are shown as individual values, with mean and standard deviation. *P*-values are from *t*-tests.

Maternal data were analyzed by maternal genotype since all were nursing the same genotype of pups; splitting into 4 groups did not reveal any differences between maternal sub-groups. Consistent with our prior studies, and despite the “rescue diet,” *Cyp27b1* null females had hypocalcemia (Figure 7A), hypophosphatemia (Figure 7B), secondary hyperparathyroidism (Figure 7C), lower FGF23 (Figure 7D), very low calcitriol (99.6 ± 14.1 pmol/L, above the 20 pmol/L detection limit due to fetal-placental sources) (Figure 7E), and higher 25(OH)D_3_ (Figure 7F). IGF-I did not differ between null and WT dams (Figure 7G). Lack of conversion of 25(OH)D_3_ into calcitriol led *Cyp27b1* null dams to significantly altered metabolite levels (24,25(OH)_2_D_3,_ 25-OH-D_3_-26,23-lactone, 3-epi-25-OH-D_3_) and increased ratios of 25(OH)D_3_ to the metabolites (25(OH)D_3_:24,25(OH)_2_D_3_, and 25(OH)D_3_:lactone) (Supplementary Figure 1). Urine calcium, phosphorus, and magnesium did not differ among the groups (Supplementary Figure 4).

**Figure 8:**
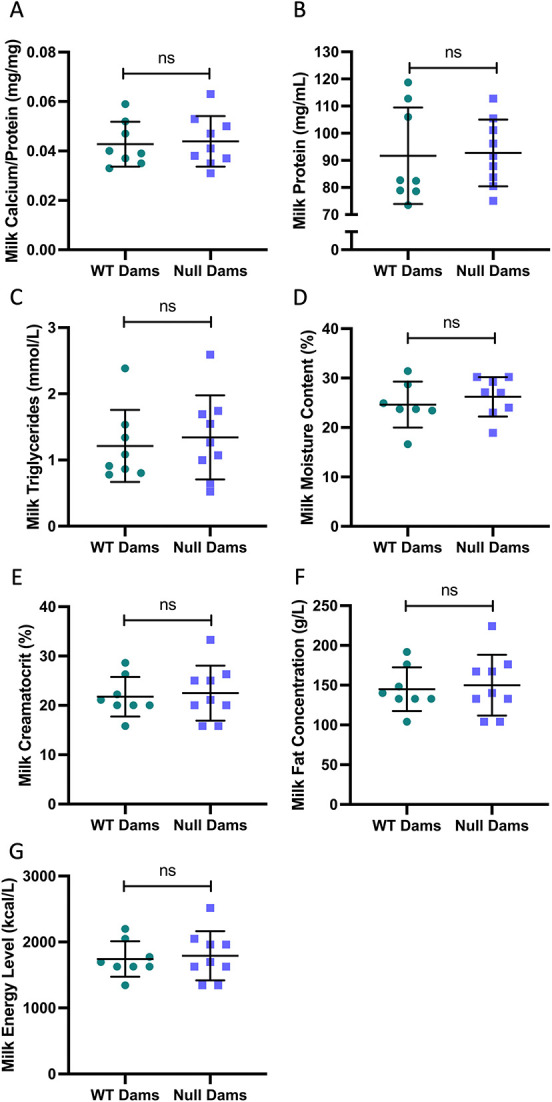
Milk composition at day 10. Milk nutritional content was no different between *Cyp27b1* null and WT, including calcium (A), protein (B), triglycerides (C), moisture (D), creamatocrit (E), fat (F), and energy (G). Data are shown as individual values, with mean and standard deviation. *P*-values are from *t*-tests.

Milk was easily sampled from both groups of mice with no evident difference in the recoverable volume. Milk nutritional content was no different between *Cyp27b1* null and WT, including measures of calcium, protein, triglycerides, moisture, creamatocrit, fat, and energy (Figure 8).

**Figure 9:**
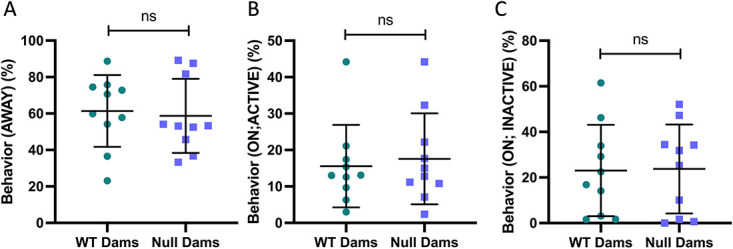
Maternal lactational behavior. During a total of 6 d of continuous video surveillance for each dam with its litter, *Cyp27b1* null and WT dams showed no differences in the percentage of time spent off the litter (A) or on the litter while active (B) or inactive (C). The inactive state, with the dam resting on the litter, is when the pups are most likely to obtain the most milk. Data are shown as individual values, with mean and standard deviation. *P*-values are from t-tests.

Maternal lactational behavior showed no differences in time spent off the litter, or on the litter while active or inactive (Figure 9).

### Resolution of offspring skeletal phenotype by day 42

**Figure 10:**
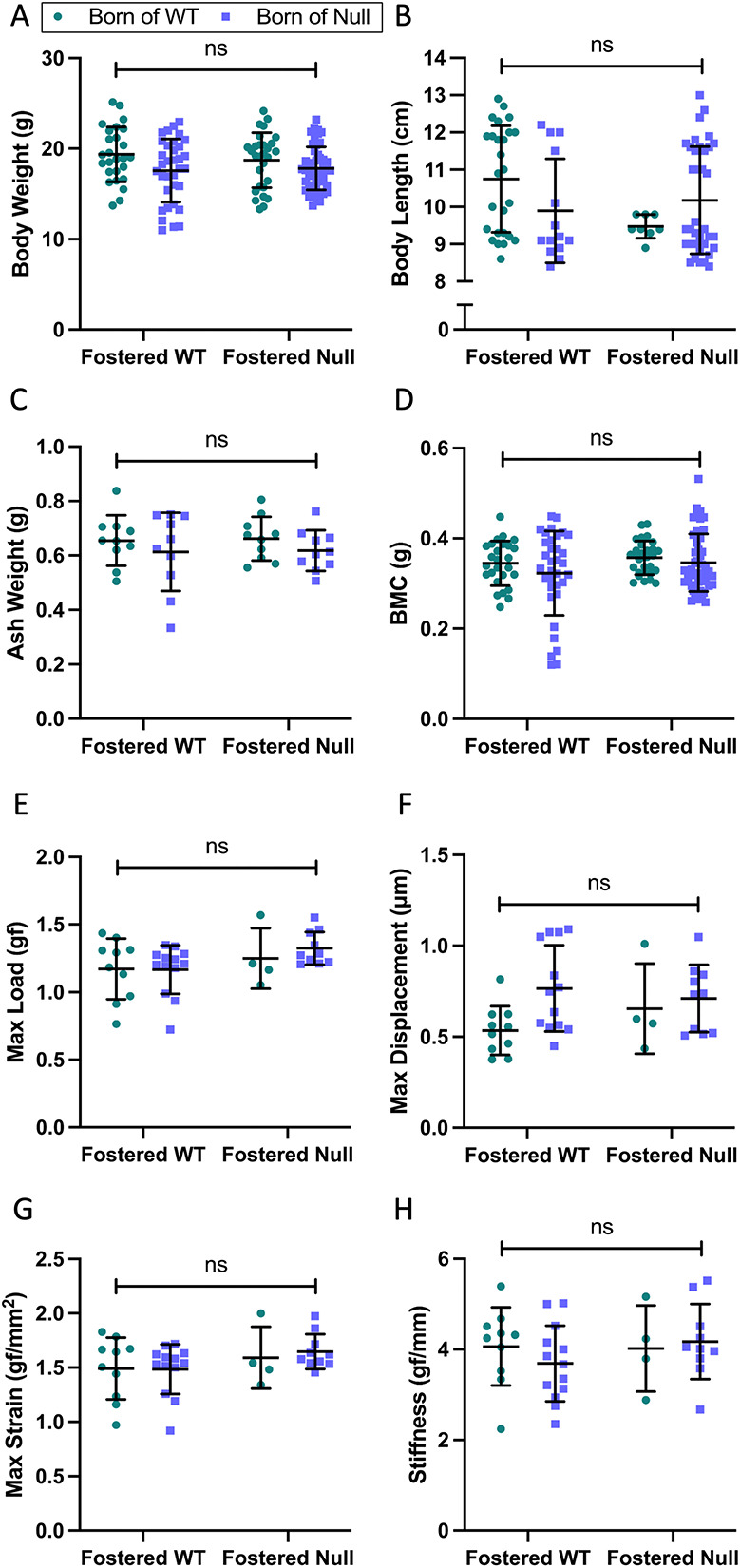
Resolution of offspring phenotype by day 42. Three weeks after weaning, and while consuming normal chow, there were no longer any between-group differences in body weight (A), body length (B), ash weight (C), and whole body BMC (D). A 3-point bend test on the tibias indicated no effects of the birth or foster dam on ultimate strength, displacement, maximum strain, and stiffness (E–H). Data are shown as individual values, with mean and standard deviation. *P*-values are from 2-way ANOVA (bold) with Šidák post hoc tests.

Additional cross-fostered pups were allowed to continue for 3 wk after weaning before being harvested at 6 wk of age (day 42). There were no between-group differences in body weight (Figure 10A), body length (Figure 10B), ash weight (Figure 10C), and whole body BMC (Figure 10D). Sex-specific analyses showed persistence of a slightly lower body weight in male pups fostered by null dams (Supplementary Figure 5A), with no effect of the null dam on male pup body length, ash weight, BMC, or BMC adjusted for body weight (Supplementary Figure 5B–E). WT → null male pups did have a higher BMC than null→null male pups. Conversely, female mice showed no between-group differences in body weight (Supplementary Figure 5A), body length (Supplementary Figure 5B), ash weight (Supplementary Figure 5C) or BMC (Supplementary Figure 5D), whereas BMC corrected for body weight was slightly but significantly higher in female pups fostered by null dams (Supplementary Figure 5E).

A 3-point bend test indicated no effect of the foster dam or birth dam on ultimate strength, displacement, maximum strain, and stiffness (Figure 10E–H). The sex-specific analysis showed similar results (Supplementary Figure 6).

## Discussion

In this study we found that, at the time of weaning (3 wk of age), offspring fostered by *Cyp27b1* null dams had lower body weight, ash weight, and whole body BMC by DXA. Their skeletons were proportionately smaller, such that adjustment for body weight attenuated the between-group differences in ash weight and eliminated the difference in whole body BMC. However, the bone was under-mineralized as indicated by disproportionately lower ash calcium and magnesium content that were not eliminated by adjusting for weight, impairment of both cortical and trabecular microarchitecture, and 3-pt bend test results that were consistent with reduced strength. There were no differences in offspring serum minerals, hormones, urine mineral excretion, or duodenal and renal gene expression to explain the lower body weight, lengths, ash weight, and mineral content. Maternal lactational behavior was no different between WT and *Cyp27b1* null dams, nor were there any differences in milk nutritional content or recoverable volume of milk. *Cyp27b1* null dams did have hypocalcemia, secondary hyperparathyroidism, absent calcitriol, higher 25OHD, and altered vitamin D metabolites. In follow-up at 6 wk of age, which is 3 wk after weaning, there were no longer any differences between offspring groups in body weight, ash weight, mineral content, whole body BMC, and biomechanical parameters of the tibias, indicating that the differences observed at weaning were recoverable.

Sex-specific analysis of male and female pups showed the same phenotype of lower body weight, shorter body lengths, and lower BMC at 3 wk of age in pups fostered by null dams, with the differences in BMC eliminated after adjustment for body weight. The relative changes in skeletal microarchitecture of pups fostered by WT vs null mice seen in the group (both sexes included) analysis were largely preserved, although statistical significance was lost in several subgroup comparisons, likely because sample numbers within each sex were insufficiently powered for such analysis. By day 42 (3 wk after weaning), in the sex-specific analysis, both sexes of pups fostered by null dams had also caught up to those fostered by WT dams in terms of lengths, ash weight, and BMC, except that male but not female pups had a slightly lower body weight when fostered by null dams. BMC was higher in WT → null compared to null→null pups but the difference was eliminated after adjustment for body weight. Conversely, although BMC was no different across groups among female pups, the adjusted BMC was higher in female pups fostered by null dams, a reversal of the finding from 3 wk of age. Ash weight is the gold standard against which DXA technology was calibrated to derive a value for BMC, and so the lack of difference in ash weight across male and female pups is more definitive. Therefore, the small differences in BMC of WT → null vs null→null pups, as well as the adjusted BMC of female pups fostered by null dams, may be chance findings due to smaller subgroups.

In our earlier study,^24^ we reported that *Cyp27b1* null and *Cyp27b1*^+/−^ fetuses from *Cyp27b1* null dams were skeletally and biochemically indistinguishable from each other, while WT and *Cyp27b1*^+/−^ fetuses from WT dams were also indistinguishable from each other. When compared across groups, fetuses of WT and *Cyp27b1* null dams had the same skeletal lengths, ash weight, and mineral content. However, fetuses of *Cyp27b1* null dams had higher serum calcium, higher amniotic fluid calcium, lower amniotic fluid phosphorus, lower serum FGF23, and higher 25OHD and 24,25(OH)_2_D, and other altered vitamin D metabolite levels as compared to fetuses of WT dams.

Those data in the earlier study imply that in this follow-up study, pups born of *Cyp27b1* null dams also began postnatal life with identical skeletal lengths, ash weight, and mineral content, but higher serum calcium, lower FGF23, and higher 25OHD and 24,25(OH)_2_D, than pups born of WT dams. The present study was designed to study maternal influences on postnatal growth without confounding from differing offspring genotypes. For example, WT, *Cyp27b1*^+/−^, and *Cyp27b1* null pups might differ in their nutritional demands and behavior. By making all pups *Cyp27b1*^+/−^, fetal and postnatal offspring demands were equalized between WT and *Cyp27b1* null dams.

The cross-fostering of pups, both to the opposite and to the same genotype as the birth dam, also enabled us to identify any prenatal influences on offspring growth and skeletal development. Largely, the offspring phenotypes were dictated by the foster dam and not the birth dam. Consequently, the biochemical differences in fetuses at the time of birth, conferred by the birth dam and observed in our earlier study, were a lesser contributor to the offspring phenotype than the foster dam’s influence.

After 3 wk of consuming the foster dam’s milk, a difference emerged with pups fostered by *Cyp27b1* null dams being globally smaller, a finding that persisted in the sex-specific analysis. The smaller skeletal size was in proportion to weight but also showed disproportionately lower mineralization (in terms of mineral content per weight of ash) and impaired microarchitecture. We sought hormonal, biochemical, and gene expression differences between the offspring that could explain the phenotype, but found no clear explanation. For example, IGF-I is a vitamin D-dependent factor that influences offspring growth. However, its level was the opposite of expected if it were to explain the reduced growth. Specifically, its value was lowest rather than highest in WT → WT pups as compared to pups fostered by *Cyp27b1* null dams. We studied maternal lactational behavior and found that WT and *Cyp27b1* null dams spent the same proportions of time on the nest versus off. We comprehensively examined milk nutritional content and the recoverable volume of milk, and found no differences to explain altered growth.

What, therefore, is the mechanism for the growth stunting that is largely attributable to the foster dam being null, with lesser influence of the birth dam? Conceivably, the milk may lack a nutrient that we did not measure, may contain higher levels of an unknown growth inhibiting factor, or may lack an anti-satiety factor. We cannot rule out these theoretical possibilities. Notwithstanding that the recoverable volume of milk did not differ between WT and *Cyp27b1* null dams, we also cannot rule out that pups fostered by null dams ingested less milk.

We chose to examine 3 wk of age because this interval is largely spent consuming the dam’s milk and because the offspring skeletons would be large enough to permit the analyses that we carried out. However, by 3 wk of age, maternal influences are already waning since the pups begin to consume solid food in the last few days before weaning. Therefore, it is possible that differences in biochemical or hormonal indices were present in the offspring at an earlier time, but attenuated or absent by 3 wk of age. It is also notable that only modest differences were seen between pups fostered by *Cyp27b1* null vs. WT dams, as compared to the strikingly abnormal maternal mineral homeostasis in adult *Cyp27b1* nulls seen both in this study and our prior studies.^37^ This implies that lactating *Cyp27b1* null dams are able to provide the necessary nutrition to their pups, despite the dams being unable to normalize their own systemic mineral homeostasis.

At 6 wk of age, which is after 3 wk of exclusively consuming regular chow, the growth restriction of pups fostered by null dams was no longer present; in the group analysis, pups had the same weight, lengths, ash weight, mineral content, whole body BMC, and skeletal strength regardless of birth dam or foster dam. This indicates that the maternal influences on postnatal growth are transient and can be reversed once the pup is consuming adult food. However, there may be sex-specific differences, since male pups fostered by null dams weighed slightly less, despite no change in length, ash weight, or BMC when compared to male pups fostered by WT dams. No differences were seen in weight, length, ash weight, or BMC at 6 wk of age between female mice fostered by WT vs null dams, although BMC adjusted for weight was higher in female pups fostered by null dams. Whether these are real differences or chance findings from analysis of smaller subgroups remains uncertain. Again, the ash weight should be considered more definitive than changes in BMC.

Could higher levels of 25OHD or its metabolite 24,25(OH)_2_D contribute to the offspring phenotype that we observed? At the time of birth, pups of *Cyp27b1* null dams had 25OHD and 24,25(OH)_2_D levels twice as high as pups of WT dams, as seen in our prior study.^24^ The higher levels in offspring birthed by null dams, achieved from free transplacental passage from the dam, will decline over the ensuing weeks; the half-life of 25OHD is 14 d in humans but has not been determined in mice.^47^ 25OHD has lower penetrance into milk, but pups fostered by *Cyp27b1* null dams will have received more 25OHD than pups fostered by WT dams; this is reflected in *Cyp27b1* null dams having 4-fold higher 25OHD (Figure 7) and pups fostered by them having a higher serum 25OHD level (Figure 5). Conversely, although the 24,25(OH)_2_D level was twice as high at birth in pups born of null vs WT dams,^24^ the 24,25(OH)_2_D level of null dams is half that of WT dams (Supplementary Figure 4), and pups fostered by null dams should receive less 24,25(OH)_2_D through milk than pups fostered by WT dams. The net effect in Table 1 showed no significant difference in 24,25(OH)_2_D among pups at 3 wk of age. Overall, null→null pups will have experienced consistently higher 25OHD levels during gestation and while nursing, null→WT and WT → null will have had intermediate exposure, and WT → WT pups will have had consistently lower 25OHD levels. Consistent with 25OHD potentially having an effect is that in the group analysis (both sexes included), body weight, body length, ash weight, ash calcium, and BMC followed an inverse pattern, being significantly higher in WT → WT pups, lowest in null→null pups, and of intermediate values in the other 2 groups.

Is there biological plausibility for 25OHD or 24,25(OH)_2_D to directly or indirectly affect growth and skeletal mineral accrual? 25OHD binds to the VDR with low activity^48^; this becomes clinically apparent only at very high 25OHD levels (>200 nmol/L) in hypervitaminosis D.^49^ At such high levels of 25OHD there is also increased conversion to 5,6-*trans-*25(OH)D_3_, which also binds to VDR with increased affinity and acts like calcitriol.^49^ The null dams had a mean 25OHD of 200 nmol/L in this study but the value in the pups was well below this. There is greater plausibility for 24,25(OH)_2_D, which has been shown to play a key role in fracture healing of mice,^50–52^ and in the maturation of chondrocytes and osteoblasts.^53^^,^^54^ Therefore, altered levels of 24,25(OH)_2_D could conceivably affect early postnatal skeletal development and mineralization, although this remains to be explored.

There are no human studies examining 24,25(OH)_2_D on fetal or neonatal skeletal parameters. However, there are human data supporting the possibility that higher 25OHD levels in the neonate may impair growth and mineral accrual (in turn, higher 25OHD may alter 24,25(OH)_2_D). Several studies have found an inverse U-shaped relationship between either late-pregnancy maternal 25OHD or cord blood 25OHD and offspring body size and weight, with higher 25OHD associated with increased risk of small-for-gestational-age babies.^55^^,^^56^ An observational study of 798 healthy mother–infant pairs found that mothers with the highest 25OHD levels during pregnancy had infants that were shorter, lighter, and leaner (weight/length) at 6 mo of age.^57^ The authors of that study speculated that higher 25OHD may slow infant growth. Among clinical trials, calcium supplementation in 69 children found that significantly lower gains in bone area and bone mineral content were achieved in children with higher 25OHD and lower PTH levels.^58^ The larger MAVIDOS trial randomized 1134 pregnant women to 1000 IU vitamin D vs placebo, and in its primary analysis, there were no differences in offspring BMC or BMD measured by 14 d after birth.^59^ However, a post hoc, unadjusted subgroup analysis showed a nominally higher (*P* = .04) BMD in winter-born babies of vitamin D-supplemented vs placebo-treated mothers, whereas in autumn-born babies of vitamin D-supplemented vs. placebo-treated mothers, the BMD was lower by a comparable magnitude (*P* = .07). Similar opposing effects were seen in the BMC response to vitamin D supplementation of winter vs. autumn-born babies. In that study, the highest maternal 25OHD levels in vitamin D-treated mothers (and greatest difference from placebo-treated mothers) were achieved in mothers whose babies were born in August through October.^59^ Taken at face value, the results are compatible with an inverse U-shaped response of BMD and BMC in newborns to the 25OHD levels they were born with, although it is also possible that these findings may be chance results attributable to small subgroups and the post hoc analyses. Many clinical studies of 25OHD’s associations with extraskeletal outcomes (such as cancer and cardiovascular mortality) have found U-shaped curves with adverse effects of lower and higher 25OHD^60^; whether these are statistical aberrations resulting from associational analyses, or represent real evidence of adverse effects of higher 25OHD, remains unresolved.

This study has multiple strengths, including use of first-degree relative dams, a rigorously controlled cross-fostering technique, equal litter sizes, and a single offspring genotype. Normalized litter sizes and one genotype of offspring meant that WT and *Cyp27b1* null dams experienced equal demands for mineral delivery during pregnancy and lactation. Conversely, multiple offspring genotypes would have created multiple subgroups and thereby reduced statistical power significantly. The *Cyp27b1* null mouse is a model not only for CYP27B1 deficiency but also vitamin D deficiency during pregnancy and lactation, since both conditions lead to inadequate calcitriol, hypocalcemia, secondary hyperparathyroidism, and other similar effects. Limitations include that the study was not powered to test sex differences in pup responses to WT vs null foster dams, leading to loss of statistical significance and possible chance results in some of the sex-specific subgroup analyses.

In conclusion, maternal loss of calcitriol impaired early postnatal growth, leading to a lower skeletal size with disproportionately reduced ash weight, and impaired cortical and trabecular microarchitectural parameters. These effects on growth, mineralization, and skeletal strength were reversed within 3 wk after the pups were no longer consuming milk, except that male mice fostered by null dams had slightly reduced body weight but not length, ash weight, or BMC at 6 wk of age.

## Supplementary Material

Supplemental_Figure_1_zjae035

Supplemental_Figure_2_zjae035

Supplemental_Figure_3_zjae035

Supplemental_Figure_4_zjae035

Supplemental_Figure_5_zjae035

Supplemental_Figure_6_zjae035

## Data Availability

Data available on request from the authors.
