# Supplementary figures and images for: Loss of maternal calcitriol reversibly alters early offspring growth and skeletal development in mice
Source: J Bone Miner Res. 2024 Mar 4;39(5):595–610. doi: 10.1093/jbmr/zjae035 (PMC11206081; doi:10.1093/jbmr/zjae035)

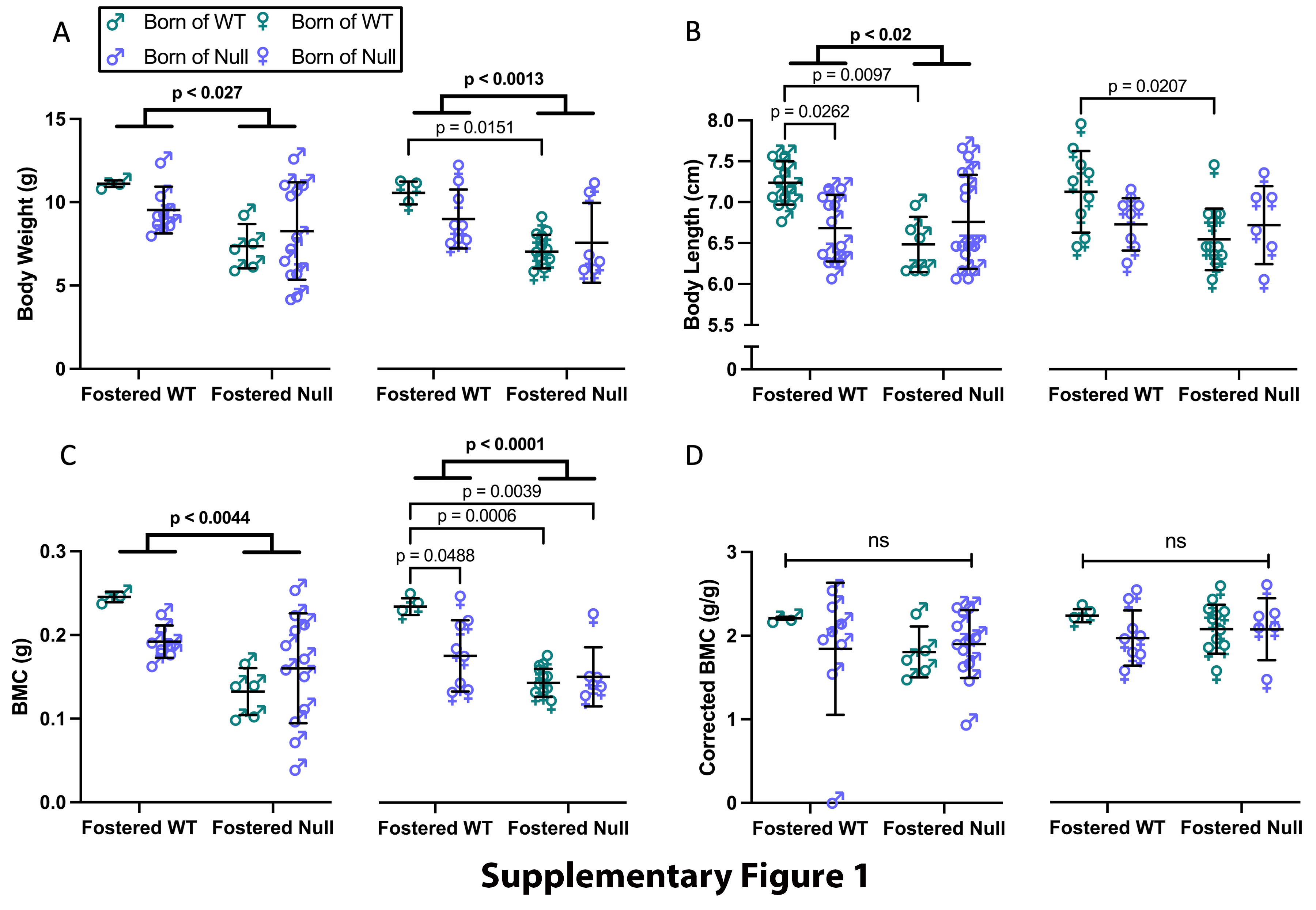

Supplement: Supplemental_Figure_1_zjae035 [file Supplemental_Figure_1_zjae035.tiff]

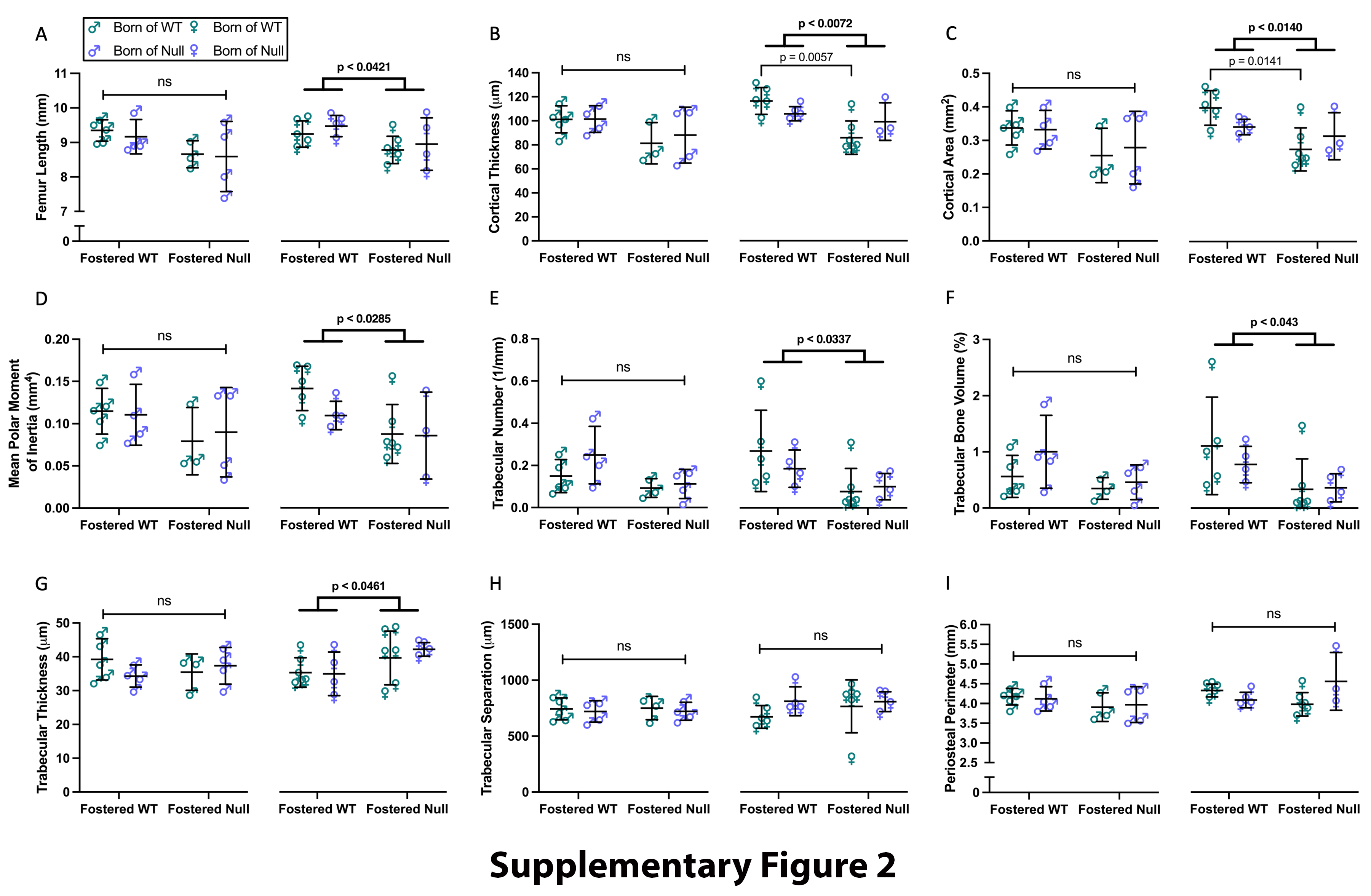

Supplement: Supplemental_Figure_2_zjae035 [file Supplemental_Figure_2_zjae035.tiff]

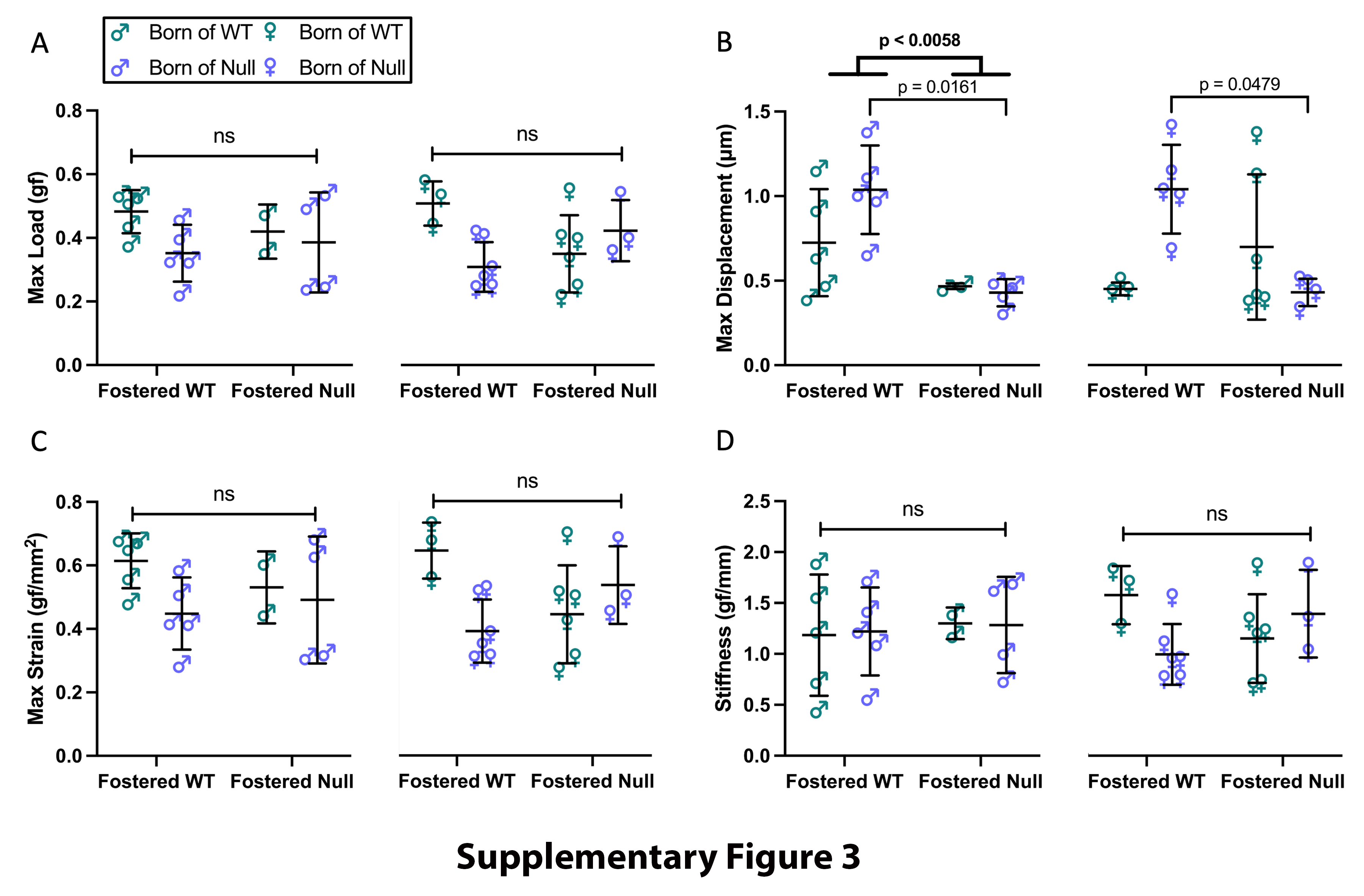

Supplement: Supplemental_Figure_3_zjae035 [file Supplemental_Figure_3_zjae035.tiff]

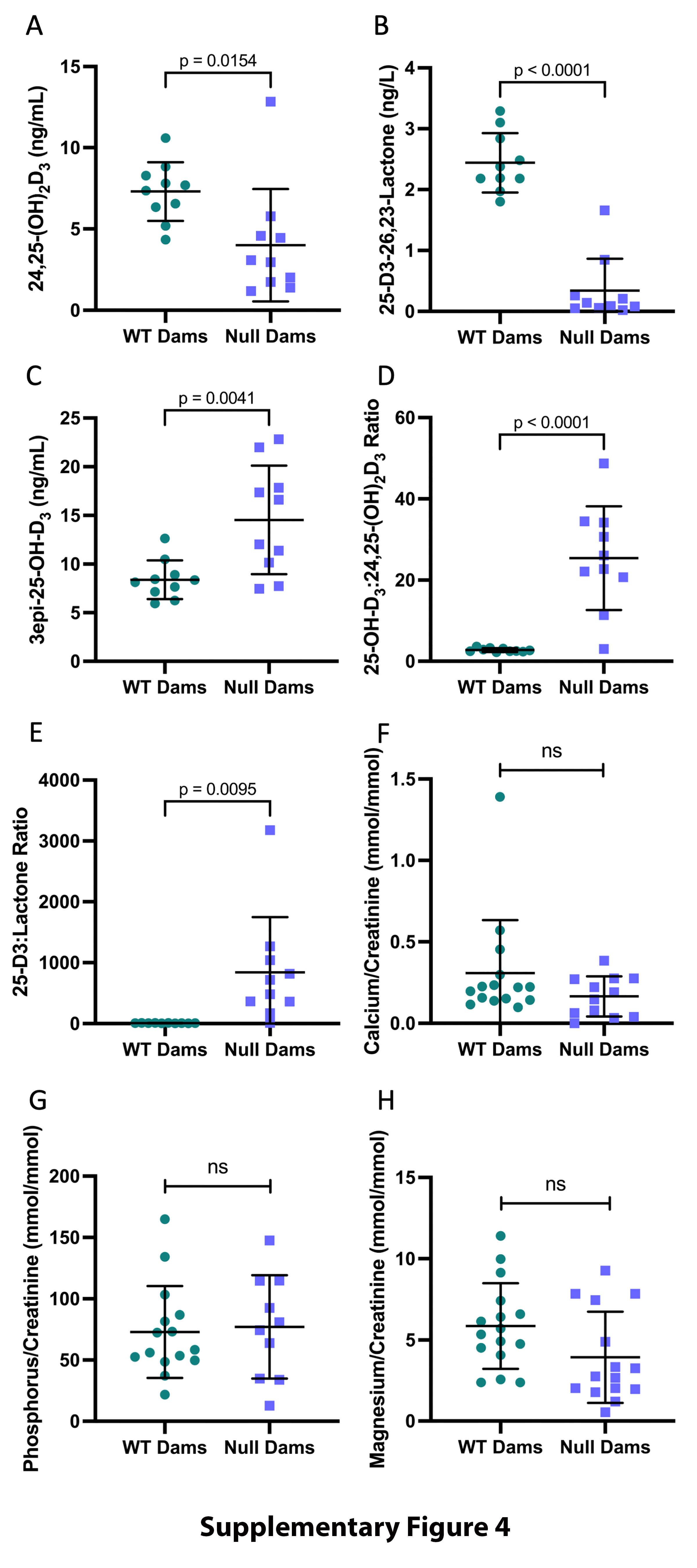

Supplement: Supplemental_Figure_4_zjae035 [file Supplemental_Figure_4_zjae035.tiff]

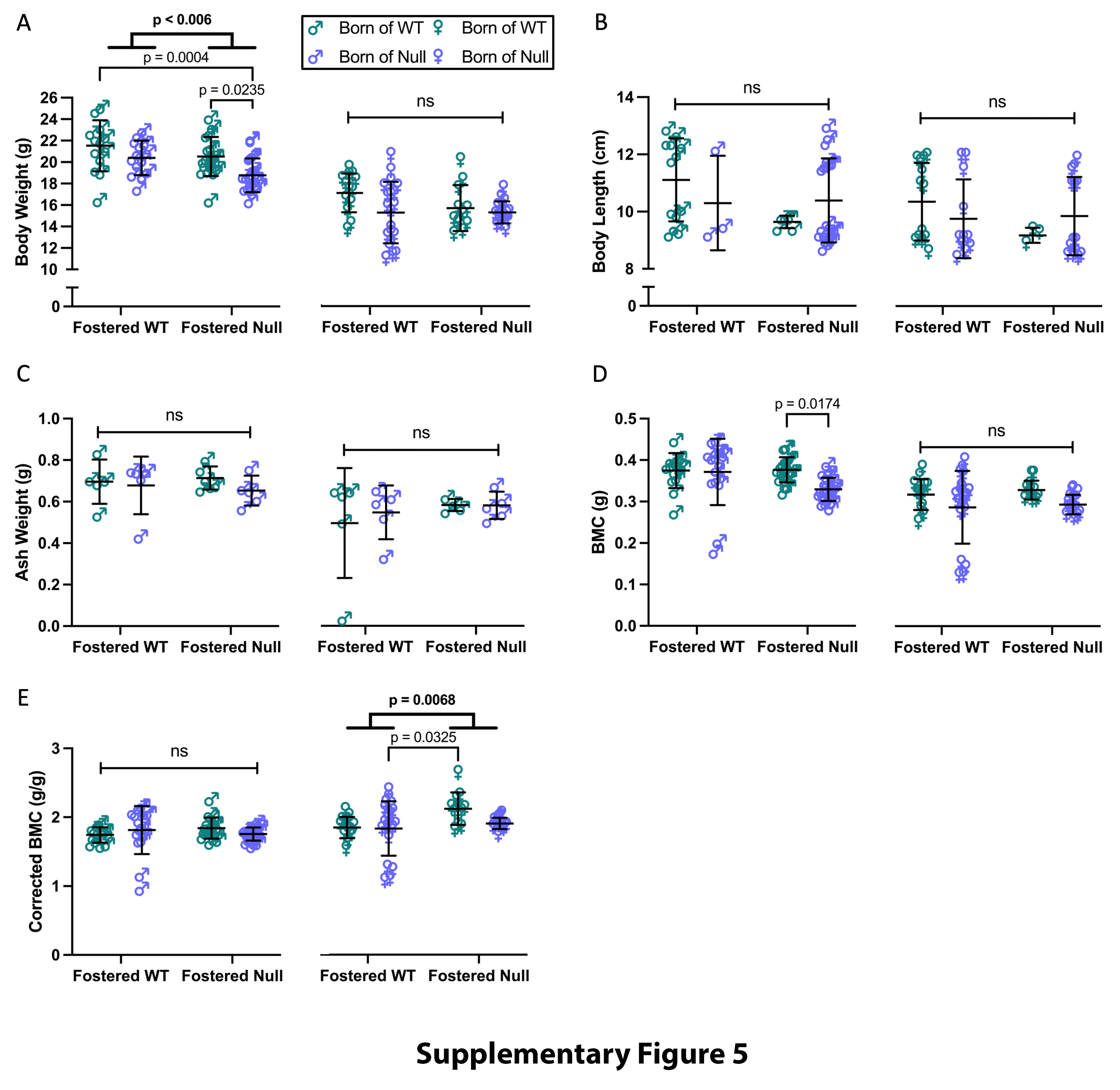

Supplement: Supplemental_Figure_5_zjae035 [file Supplemental_Figure_5_zjae035.tiff]

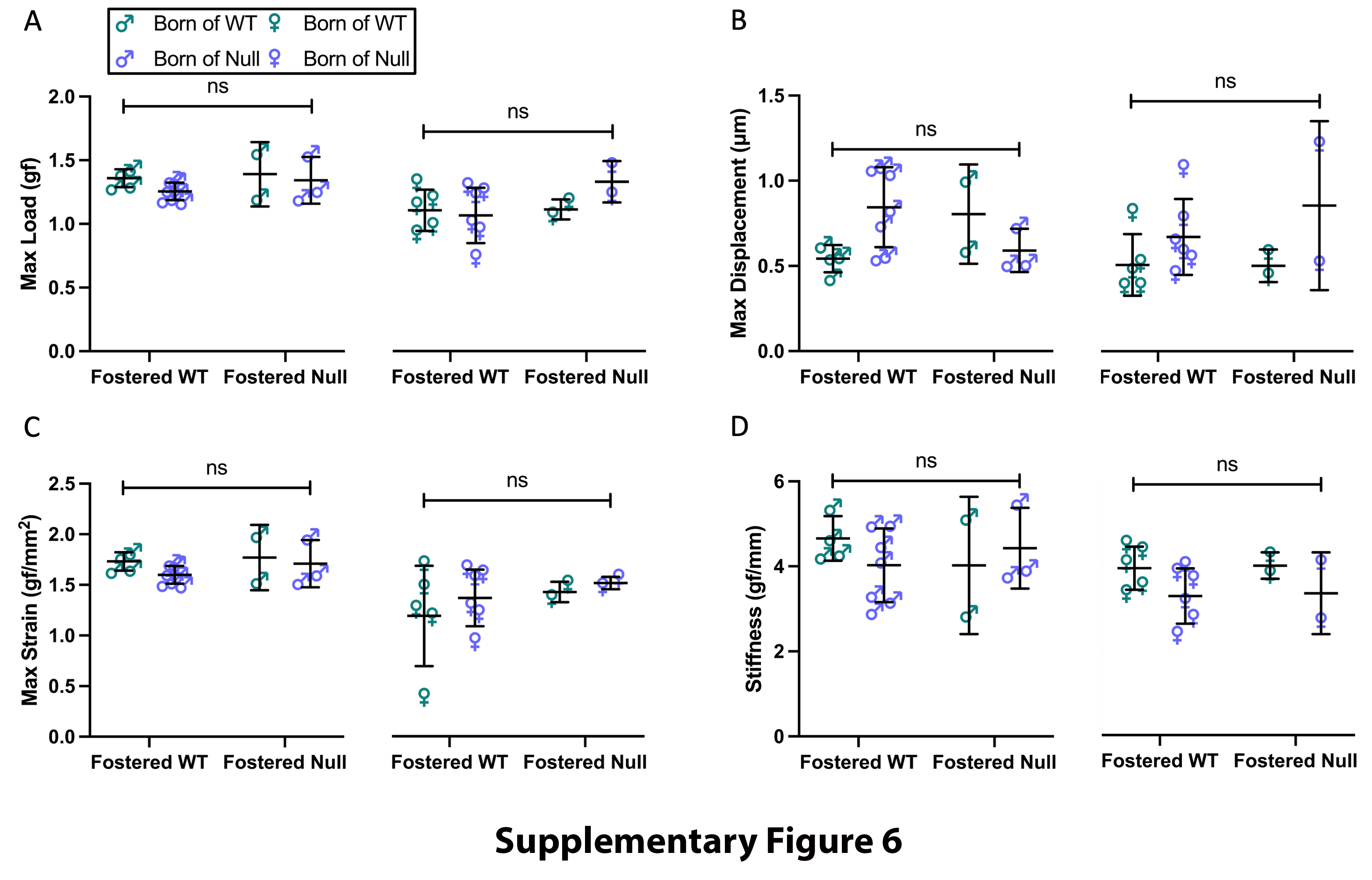

Supplement: Supplemental_Figure_6_zjae035 [file Supplemental_Figure_6_zjae035.tiff]
